# New Insights into the Cell Death Signaling Pathways Triggered by Long-Term Exposure to Silicon-Based Quantum Dots in Human Lung Fibroblasts

**DOI:** 10.3390/nano11020323

**Published:** 2021-01-27

**Authors:** Miruna S. Stan, Smaranda Badea, Anca Hermenean, Hildegard Herman, Bogdan Trica, Beatrice G. Sbarcea, Anca Dinischiotu

**Affiliations:** 1 Department of Biochemistry and Molecular Biology, Faculty of Biology, University of Bucharest, 91-95 Spl. Independentei, 050095 Bucharest, Romania; miruna.stan@bio.unibuc.ro (M.S.S.); smara.badea@gmail.com (S.B.); anca.hermenean@gmail.com (A.H.); 2 Research Institute of the University of Bucharest–ICUB, University of Bucharest, 91-95 Spl. Independentei, 050095 Bucharest, Romania; 3 Department of Science and Engineering of Oxide Materials and Nanomaterials, Faculty of Applied Chemistry and Materials Science, University Politehnica of Bucharest, 1-7 Gheorghe Polizu Str., 011061 Bucharest, Romania; 4 Institute of Life Sciences, Vasile Goldis Western University of Arad, 86 Rebreanu, 310414 Arad, Romania; hildegard.i.herman@gmail.com; 5 National Institute for Research & Development in Chemistry and Petrochemistry (INCDCP-ICECHIM), 202 Spl. Independentei, 060021 Bucharest, Romania; trica.bogdan@gmail.com; 6 Materials Characterization Department, National Institute for Research & Development in Electrical Engineering (ICPE-CA), 313 Splaiul Unirii, 030138 Bucharest, Romania

**Keywords:** cell death, redox signaling, apoptosis, autophagy, telomeres, quantum dots, lung fibroblasts

## Abstract

This report is the first research study that aims to explore the molecular mechanisms involved in the in vitro pulmonary cytotoxicity triggered by long-term exposure to silicon-based quantum dots (QDs). Human lung fibroblasts (MRC-5 cell line) were exposed to 5 µg/mL silicon-based QDs for 5 weeks and the concentration was increased up to 40 µg/mL QDs during the next 4 weeks. Cell viability and population doubling level were calculated based on Trypan blue staining. The expression levels of proteins were established by Western blotting and the telomeres’ length was determined through Southern blotting. Prolonged exposure of lung fibroblasts to QDs reduced the cell viability by 10% compared to untreated cells. The level of p53 and apoptosis-inducing factor (AIF) expression increased during the exposure, the peak intensity being registered after the seventh week. The expressions of autophagy-related proteins, Beclin-1 and LC-3, were higher compared to untreated cells. Regarding the protein expression of Nrf-2, a progressive decrease was noticed, suggesting the downregulation of a cytoprotective response to oxidative stress. In contrast, the heat shock proteins’ (HSPs) expression was increased or maintained near the control level during QDs exposure in order to promote cell survival. Furthermore, the telomeres’ length was not reduced during this exposure, indicating that QDs did not induce cellular senescence. In conclusion, our study shows that silicon-based QDs triggered the activation of apoptotic and autophagy pathways and downregulation of survival signaling molecules as an adaptive response to cellular stress which was not associated with telomeres shortening.

## 1. Introduction

A promising approach for in vivo imaging, tumor biology investigation and cancer treatment is based on the use of quantum dots (QDs). Although these nanometer-scale semiconductor crystals have multiple optical and electronic properties which provide improved advantages for medical purposes compared to conventional nanoparticles (NPs), their characteristics can also trigger toxicity effects in healthy cells by different mechanisms, as it was recently reviewed by Zhu et al. [1].

The efficiency of QDs applications especially involves their internalization inside the cells, this process being directly influenced by the characteristics of NPs (such as shape, size, load, functionalization and protein corona) [2], the type of cells that can express certain receptors and the phases of the cell cycle [3]. An efficient internalization was noticed for spherical particles with a radius between 20 and 30 nm, where the agglomeration of NPs could promote the uptake [4]. Researchers observed a caveolae-mediated endocytosis in NPs below 20 nm and clathrin-mediated endocytosis and macropinocytosis for particles with sizes higher than 40 nm [5]. For very small QDs (such as 4 nm), it was shown that they first attach to the cell surface and a complete membrane wrapping occurs only if a large cluster of particles are packaged in one vesicle as they cannot be taken up individually [6]. Due to the negative charge of the cell membrane glycocalyx, positively charged NPs have a higher efficiency of electrostatic interaction with the membrane and thus a higher rate of internalization compared to neutral or negatively charged NPs due to the membrane’s tendency to maintain its initial charge distribution [7].

Furthermore, the adsorption of different biomolecules from the physiological fluids can modulate the internalization of NPs, but there are contradictory data in this regard. Some studies have suggested that the presence of a protein corona facilitated interaction with the cell membrane and particle internalization [8], and others have shown a decrease in the rate of internalization in the presence of serum proteins due to decreased nonspecific interactions between the cell membrane and NPs [9]. Further, a prior coating with apolipoproteins ApoA4 and ApoC3 could decrease the uptake rate, and the overcoating with ApoH amplified it [10].

Compared to global cytotoxicity evaluation, there are few studies that have investigated the mechanism of QDs genotoxicity. It has been shown that CdTe QDs have the ability to induce global hypoacetylation of histone H3 in a concentration-dependent manner, as well as nucleus shrinkage, chromatin condensation and loss of mitochondrial crystals [11]. Graphene-based QDs also induced DNA damage reflected by an increased expression of p53, Rad1 and OGG1 (8-oxoguanine DNA glycosylase-1) proteins after exposure of NIH-3T3 cells to concentrations above 50 µg/mL QDs [12]. Oxidative DNA damage induced by particles can further contribute to telomere shortening, a process associated with cellular senescence. Due to the high content of guanine, telomeres are very sensitive to oxidative stress [13], with it being shown that senescent cells contain 30% more oxidized guanosine (8-oxo-dG) [14], mainly due to the fact that telomeric DNA is deficient in repairing single-strand breaks. In the absence of telomerase activity, it has been postulated that telomeres could be elongated by the alternative lengthening of telomeres (ALT) mechanism which could be activated in normal cells under chronic stress and not only in immortalized cells [15]. However, regarding the toxic effect induced by QDs on telomeres, it has not yet been studied in terms of their effects on length and integrity, but rather only to highlight biological and medical applications of synthesized QDs throughout the detection and measurement of telomeres’ length [16].

In the long term, the exposure of cells to the action of QDs can also affect their proliferative capacity, influencing the processes of regulating the replication and repair of genetic material. Therefore, the investigation of cellular and molecular effects induced by QDs over a longer period of time becomes a necessity in the context of their use in various biomedical applications and may provide new evidence on the dynamics of processes involved in the cytotoxicity of this particular type of nanomaterial. To the best of our knowledge, the present study assessed, for the first time, the effects triggered in vitro by Si/SiO_2_ QDs during a long-term exposure on human lung fibroblasts, since almost all of the in vitro reports include only a short-term analysis of QDs toxicity. The novelty of the present work is given also by the repeated exposure of cells to QDs that was performed in order to reveal the adaptive response related to the length of telomere sequences. MRC-5 cells have been selected to be used within this study as they are frequently used for the assessment of pulmonary cytotoxicity of nanoparticles. In addition to BEAS-2B, A549 and Calu-3, the MRC-5 cell line represents a very good choice of model to mimic in vitro a repeated exposure scenario on long-term studies regarding the potential effects of nanoparticles on lungs [17,18].

Cell viability and population doubling were monitored along with morphological alterations of human lung fibroblasts during the 9 weeks of incubation with QDs. The effect of these particles on the dynamics of cell death was analyzed throughout the expression of proteins involved in the activation and regulation of apoptosis and autophagy (evaluation of Nrf2 expression and heat shock proteins as markers of oxidative stress and regulators of various pro-survival signaling pathways, evaluation of p53, MDM2, AIF and caspases 3 and 9 expressions as indicators of apoptosis and evaluation of Beclin-1 and LC3 protein expression as indicators of autophagy). Further, the effect induced by Si/SiO_2_ QDs on the length of telomeric fragments was investigated and correlated with the other initiated cellular responses.

## 2. Experimental Section

### 2.1. QDs Synthesis and Characterization

The Si/SiO_2_ QDs tested in this study were supplied by the Laser Department of the National Institute for Laser, Plasma and Radiation Physics, Bucharest-Magurele, Romania. The nanoparticles were made of a crystalline silicon core covered by an amorphous SiO_2_ shell of 1–1.5 nm thick and were synthesized by pulsed laser ablation (using a neodymium-doped yttrium aluminum garnet (Nd:YAG) laser (EKSPLA, Vilnius, Lithuania) with the following parameters: 355 nm wavelength, pulse duration—5 nanoseconds, repetition rate—10 Hz, and energy per pulse—60 mJ) that yielded particles with an average size of 6–8 nm [19,20]. QDs showed red photoluminescence with a maximum intensity at about 644 nm when they were excited in ultraviolet (UV) light at 325 nm [20].

For TEM analysis, small drops of QDs dispersed in distilled water were put on top of a pure carbon film copper grid (Ted Pella, Inc., Redding, CA, USA). Each time, the excess was carefully removed using filter paper. The sample was analyzed in bright-field mode using a Tecnai F20 G2 TWIN Cryo-TEM (FEI, Hillsboro, OR, USA) at an acceleration voltage of 200 kV and also subjected to energy-dispersive X-ray spectroscopy (EDXS) (Oxford Instruments, Abingdon, UK).

SEM investigations were performed on a Zeiss Auriga microscope (Carl Zeiss, Jena, Germany). Images were acquired at a voltage of 2 kV and contrast-enhanced for a better examination.

The measurement of the hydrodynamic size and zeta potential of QDs dispersed in ultrapure Milli-Q water at a concentration of 100 µg/mL was performed in triplicate at 25 °C with a refractive index of 1.52 using a Malvern Nano-ZS instrument (Malvern Instruments, Malvern, Worcestershire, UK).

### 2.2. Cell Culture and Long-Term Exposure to QDs

The MRC-5 cell line was obtained from American Type Cell Culture (ATCC; Manassas, VA, USA; catalog number: CCL-171) and was maintained at 37 °C in a humidified atmosphere with 5% CO_2_. The growth medium was Minimum Essential Medium (MEM; Gibco, Grand Island, NY, USA) supplemented with 10% fetal bovine serum (MEM; Gibco, Grand Island, NY, USA) and 1% antibiotics (100 U/mL penicillin, 100 µg/mL streptomycin and 0.25 µg/mL amphotericin B; Sigma-Aldrich, St. Louis, MO, USA) and was changed every 3 days until 80% confluence was reached. Cell detachment was performed with a solution containing 0.25% trypsin and 0.53 mM ethylene diamine tetraacetic acid (Sigma-Aldrich, St. Louis, MO, USA).

A stock solution of QDs (1 mg/mL) was sonicated with Hielscher UP50H (Hielscher GmbH, Teltow, Germany) for 5 min at room temperature and sterilized by autoclaving for 20 min at 120 °C. The experiments performed in this long-term study were performed starting from cells at passage number 21, as delivered by ATCC, and seeded in 75 cm^2^ flasks at an initial density of 1 × 10^6^ cells/flask. This cell density and weekly splitting were chosen as it was proved that they can yield a population doubling level of about 1.2 after 6–7 days in adherent T-flask cultures [21]. The QDs were added in the culture media at 24 h after each seeding as we did not want to affect the adhesion of the cells on the flask’s surface. When culture medium changed (once each week), the nanoparticles were added again in the fresh culture media. The passage of cells was performed weekly, the split ratio being 1/3. The cells were exposed to 5 μg/mL Si/SiO_2_ QDs in the first 5 weeks, then the concentration of QDs was progressively increased until week no. 8 (week 6: 10 μg/mL QDs, week 7: 20 μg/mL QDs and week 8: 40 μg/mL QDs) and maintained to week no. 9. In parallel, cells were cultured under the same conditions without adding QDs to the culture medium, which served as a control for the experiments. The cell morphology was examined under the inverted optical microscope Olympus IX71 (Olympus, Tokyo, Japan).

### 2.3. Trypan Blue Staining

At the initial cell seeding step and at the end of each week of QDs incubation in the long-term experiment, MRC-5 cells were counted using the Bürker-Türk counting chamber with Trypan blue dye (Gibco, Grand Island, NY, USA) which is permeable to dead cells and stains them blue.

The percentage of cell viability was calculated weekly after each passage according to the following formula:

% viable cells = [1.00 − (Number of blue cells ÷ Number of total cells)] × 100.(1)

In order to calculate the number of population doublings (PD) only for this long-term experiment, starting from zero, without taking into account the doubling level of the inoculum used to initiate the subculture, the following formula was used [22]:

PD = log (number of cells collected/number of cells seeded)/log (2).(2)

### 2.4. TEM Analysis of Cell Samples

Cells harvested at the end of week nos. 1, 3, 5, 7 and 8 were fixed in glutaraldehyde, washed in 0.1 M phosphate buffer and post-fixed in 2% osmic acid prepared in 0.15 M phosphate buffer. The dehydration was carried out in acetone and then the samples were included in an Epon 812 resin. Ultra-sections of 60 nm thickness were made with a Leica EM UC7 ultramicrotome (Leica Microsystems GmbH, Wetzler, Germany) and analyzed on an electron microscope with a Tecnai 12 Biotwin transmission (FEI company, Eindhoven, The Netherlands).

### 2.5. Western Blot

In order to obtain the cell lysate necessary to determine the protein concentration and to perform the Western blot technique, the cells were collected weekly from one T75 flask starting with week no. 2 and washed in PBS. After centrifugation (250× *g* for 10 min), the supernatant was discarded and the cells taken from each flask were resuspended in 300 μL PBS. The cell membranes were disrupted by a sonication process performed 3 times for 30 s on ice using the Hielscher UP50H sonicator (Hielscher GmbH, Teltow, Germany). The samples were then centrifuged for 10 min at 3000× *g* to remove cell debris and the obtained supernatant containing the total protein extract was collected and stored at −80 °C. The protein concentration was assessed by Lowry’s method using the Folin–Ciocalteu reagent and the bovine serum albumin for the calibration curve.

The protein levels were quantified by Western blot using the protein supernatants collected as described above. Proteins were separated (40 µg/well) by sodium dodecyl sulphate-polyacrylamide gel electrophoresis (SDS/PAGE, 10% polyacrylamide) under reducing conditions and transferred onto 0.4 µm poly(vinylidene difluoride) membrane (Millipore, Billerica, MA, USA) in a wet transfer system (Bio-Rad, Hercules, CA, USA). The next steps (membrane blocking, incubation of antibodies, with chromogen solution) were performed using the WesternBreeze Chromogenic kit (Invitrogen, Themo Fischer Scientific, Waltham, MA, USA) and the membranes were processed according to manufacturer’s instructions. Primary mouse polyclonal antibodies against β-actin, p53, MDM2, Beclin-1, LC3-I and LC3-II, Nrf2, AIF, active caspases 3 and 9, Hsp60, Hsp70 and Hsp90 (dilution 1:250; Santa Cruz Biotechnology, Santa Cruz, CA, USA) were used for Western blot. The obtained bands were visualized with the Chemidoc MP system (Bio-Rad, Hercules, CA, USA) and quantified using ImageLab software (Bio-Rad, Hercules, CA, USA). Each sample tested was normalized to the expression corresponding to the β-actin band used as a control of protein loading.

### 2.6. Southern Blot

The genomic DNA of cells harvested at the end of week nos. 3–8 was isolated using the Wizard*^®^* Genomic DNA Purification Kit (Promega, Madison, WI, USA) according to the manufacturer’s instructions. At the end of this process, the DNA concentration was measured with NanoDrop BioSpec Nano (Shimadzu BioTech, Kyoto, Japan). All steps necessary to determine the length of the telomeric fragments were performed using the TeloTAGGG Telomere Length Assay Kit (Roche, Germany) following the indications provided by the manufacturer. After spreading, in the dark, the substrate solution (~40 drops) on the membrane’s surface, the hybridization bag was sealed using an electric sealer without air bubbles, the excess substrate solution being removed before the sealing. Membrane was incubated for 5 min and then was exposed to the chemiluminescence detection device (G:Box Chemi XR5; Syngene, Cambridge, UK) using the GeneSys software (Syngene, Cambridge, UK). Telomeric-specific smear on the Southern blot image was converted into mean terminal restriction fragments length (TRF) with the help of Image J software (version 1.49o, NIH, Bethesda, MD, USA) and the macro file MolWt.txt. TRF length of each lane containing a different DNA sample was analyzed separately using Image J software on the basis of migration distance of telomeric DNA in comparison to the known molecular weight (MW) DNA marker provided by the kit. Each sample lane of the scan image was overlaid with a grid of 32 squares of equal length. For each square that contains DNA, the optical density or pixels’ intensity (*OD_i_*) was determined and also the corresponding length or molecular weight at the mid-point of that square (*L_i_*) based on the MW DNA marker. Mean TRF length was defined according to the following formula:

(3) $$\bar{TRF}=\;\frac{\sum \left(OD_{i}\right)}{\sum \left(\frac{OD_{i}}{L_{i}}\right)}$$

### 2.7. Statistical Analysis

The statistical significance of results was analyzed by Student’s *t*-test (Microsoft Excel, Microsoft, Redmond, WA, USA). Data are calculated as mean ± standard deviation (SD) from three independent experiments and presented as a percentage of the control mean. A value of *p* less than 0.05 was considered to be statistically significant.

## 3. Results and Discussion

### 3.1. Characterization of Si/SiO_2_ QDs

The morphology and physicochemical properties of Si/SiO_2_ QDs were characterized by transmission and scanning electron microscopy (TEM and SEM, respectively), energy-dispersive X-ray spectroscopy (EDXS) and hydrodynamic size and zeta potential measurements.

**Figure 1:**
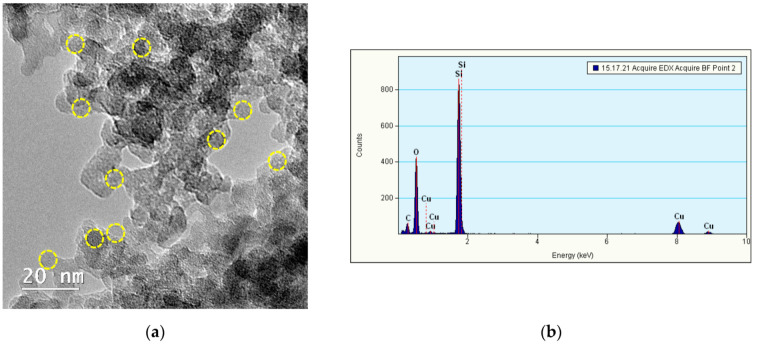

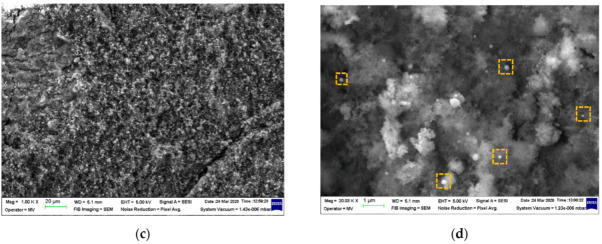
Characterization of Si/SiO_2_ quantum dots (QDs) by TEM (**a**), EDXS (**b**) and SEM (scale bar of 20 µm (**c**) and scale bar of 1 µm (**d**)) investigations. Note the spherical shape of QDs marked by yellow dot circles (**a**) and the presence of individualized spherical silicon mono-crystals of nanometer sizes marked by orange dot squares (**d**).

Although the QDs have a high tendency to aggregate, with the observed particles being closely packed, the TEM investigation confirmed the predominant spherical shape of QDs (Figure 1a—yellow dot circles). Further, their size was lower than 10 nm, with a noticeable crystallin silicon core covered by a fine shell (≤2 nm) of amorphous silica, as it was described before [19]. The EDXS analysis (Figure 1b) showed the presence of silicon and oxygen as part of the Si/SiO_2_ QDs structure, confirming the pure composition of Si/SiO_2_ particles, the carbon and copper content being part of the carbon holey copper grids used for TEM determinations. Taking into account the high degree of QDs aggregation, a SEM investigation of the aggregates’ surface topography was absolutely necessary for a further correlation with the impact on cells. The SEM image of 1000× magnification (Figure 1c) provided an overview of the QDs arrangement, with a very compact topography characterized by nano- and micro-roughness being obtained. A more detailed view on SEM (20,000× magnification) showed the presence of individualized spherical Si mono-crystals of nanometer sizes (Figure 1d—orange dot squares).

The dispersion in water of Si/SiO_2_ QDs provided a hydrodynamic diameter of 190 ± 58 nm (the average value ± standard deviation (SD) for three measurements by dynamic light scattering (DLS)), reflecting the tendency of these particles to form aggregates, as it was also shown in TEM and SEM images. Furthermore, the zeta potential was −14 ± 0.8 mV (the average value ± SD for three measurements), confirming a negative electric charge on the QDs’ surface, and a poor colloidal stability correlated with their tendency to form aggregates.

### 3.2. Analysis of MRC-5 Cells’ Growth during the Long-Term Exposure to QDs

**Figure 2:**
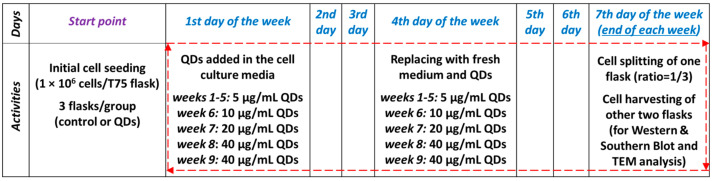
Schematic representation of the long-term exposure (9 weeks of 7 days each) of MRC-5 cells to Si/SiO_2_ QDs. The human lung fibroblasts were cultivated on three flasks of 75 cm^2^ per group (control or QDs) at an initial density of 1 × 10^6^ cells/flask (“Start point”). The QDs were added in the culture media at 24 h after each seeding (“1st day of the week”) in order to maintain a proper cell adhesion on the flasks’ surface. When culture medium was changed (“4th day of the week”), the QDs were added again in the fresh culture media. The passage of cells cultured in one flask was performed at the 7th day of the week (“the end of each week”), the split ratio being 1/3. The cells from the other 2 flasks were collected for Western and Southern blots and for TEM analysis.

The toxicity of the 5 µg/mL QDs suspension was tested weekly for 5 weeks, then the concentration used was increased to 10, 20 and 40 μg/mL over the next 3 weeks as described in Figure 2. The chosen exposure intervals allowed the establishment of QDs’ potential to induce chronic toxicity on MRC-5 cells, but also the evaluation of long-term changes, which is necessary to highlight the mechanisms of regulation of cellular functions in response to an induced stress. Our purpose was to use low concentrations of QDs, with significantly decreased toxicity, in order to be more appropriate for a realistic human exposure and clinical usage on humans. However, taking into account that human health studies on QDs are scarce, it is impossible to translate exactly the concentrations used within this work to a real human exposure. In the first 7 weeks of the experiments, concentrations below or equal to 20 µg/mL of QDs suspension were selected to be tested based on the previous results obtained for the same type of nanoparticles on short periods of incubation [19] and other particles on long-term exposure [23] that confirmed their low toxicity. Repeated exposure of cells to a low dose represents a common practice to study the potential long-term effects of nanoparticles [17]. As no dramatic changes were noticed during the first 5 weeks of exposure, we decided to gradually increase the concentration of QDs and to observe if modifications appear.

**Figure 3:**
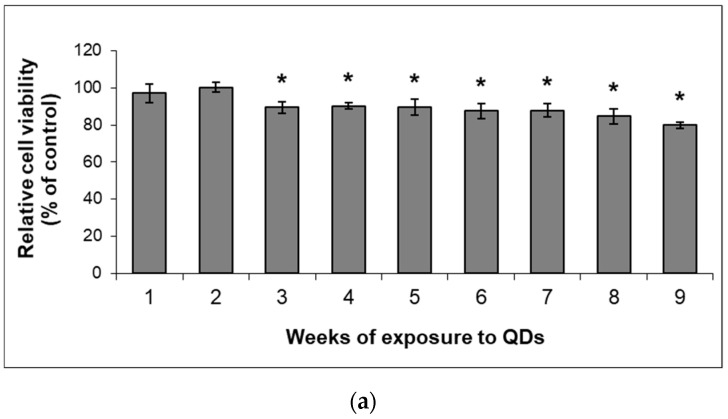

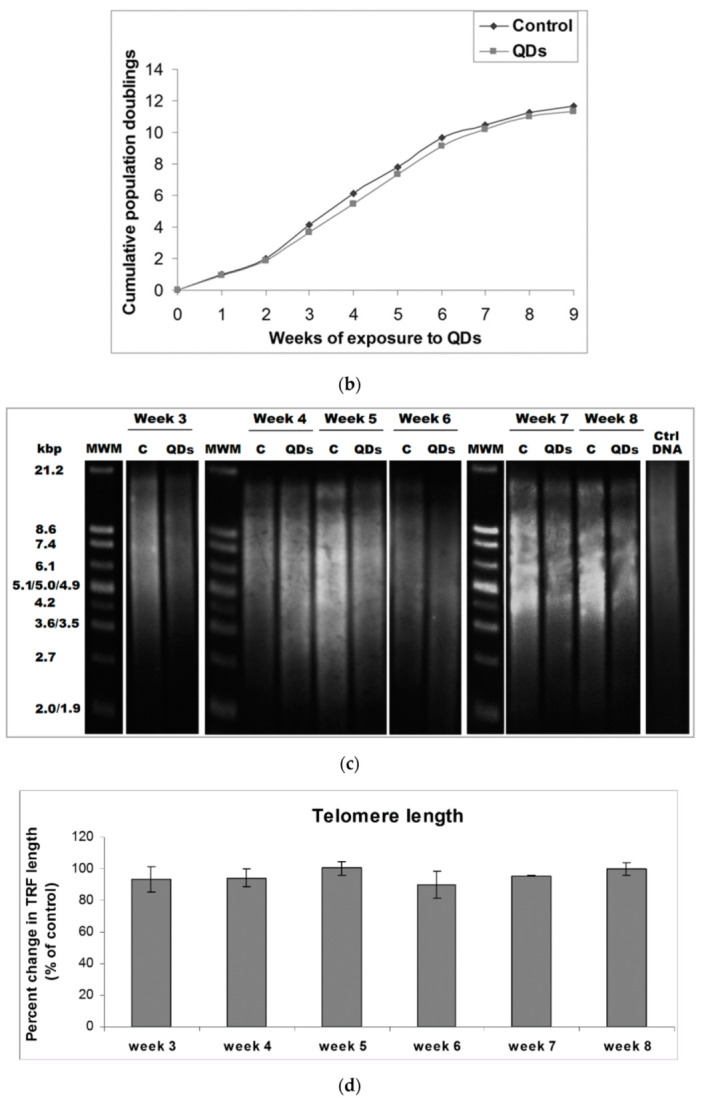
Influence triggered by Si/SiO_2_ QDs on the cell growth of MRC-5 human lung fibroblasts during the 9-week exposure. Cell viability (**a**) and number of cumulative population doublings (**b**) were determined using Trypan blue staining during the 9-week incubation of MRC-5 cells with Si/SiO_2_ QDs. Length of telomeric restriction fragments (TRFs) visualized by chemiluminescence detection (**c**) within the Southern blot technique was quantified (**d**) based on the migration distance of telomeric DNA in comparison to a known molecular weight DNA marker (MWM). The control DNA supplied by the manufacturer was represented by purified genomic DNA from immortal cell lines and confirmed that all steps of this method worked well. Results are calculated as mean ± standard deviation (SD; *n* = 3) and expressed relative to control (* *p* < 0.05 versus control).

In order to determine how cell proliferation was affected by QDs exposure, the MTT test was performed. It was found that long-term exposure to QDs did not induce a significant decrease in cell viability. A decrease of no more than 5% of control was observed after the incubation with a concentration of 5 µg/mL QDs, and no more than 20% for subsequent concentrations (Figure 3a).

During the entire period of exposure to QDs, the main stages of cell growth were observed: the lag phase, the exponential phase and the plateau phase. Analysis of the number of cumulative population doublings during the 9-week incubation of fibroblasts with QDs showed that there were no significant changes compared to control cells, with only a slight decrease during the exponential phase (Figure 3b).

To track the influence of QDs on proliferative capacity and on the induction of replicative senescence in MRC-5 cells, the telomeric fragments were detected and their lengths were quantified by Southern blot using the TeloTAGGG Telomere Length kit (Figure 3c,d). A key factor considered in this study was the number of population doublings, as the telomeric sequence is known to shorten with each cell replication. Thus, after a certain number of population doublings, adult cells reach the Hayflick limit, the threshold at which they no longer replicate and enter senescence [24]. MRC-5 cells can achieve an average of 50 population doublings until the Hayflick limit is reached [25]. In this way, it can be observed that a shortening of telomeres in the control cells in the last 3 weeks occurs (Figure 3d), which corresponded to a lower replication rate highlighted by the phase plateau of the growth curve in the last weeks of the experiment (Figure 3b). Compared to the control, the highest decrease during the whole period of incubation with QDs was by 10% after the sixth week, suggesting that the concentrations tested did not affect the length of telomeres in MRC-5 cells. Further, no changes were obtained after week 9 compared to the previous one (data not shown).

The use of the sub-culture method in long-term in vitro experiments is supported by previous studies in which it has been shown that other growth methods, such as the use of small bioreactors, may have disadvantages due to the lack of reproducibility [26]. In the present case, the technique of sub-culturing the cells after each week of exposure was used to avoid reaching cell densities that inhibit growth, as well as lack of nutrients, accumulation of residual products or changes in the pH of the environment. Our results showing that Si/SiO_2_ QDs did not affect the viability and growth rate of lung fibroblasts are consistent with those presented by Comfort and colleagues [27] that investigated the long-term exposure of keratinocytes to silver NPs.

### 3.3. QDs Internalization into MRC-5 Cells during the Long-Term Exposure

**Figure 4:**
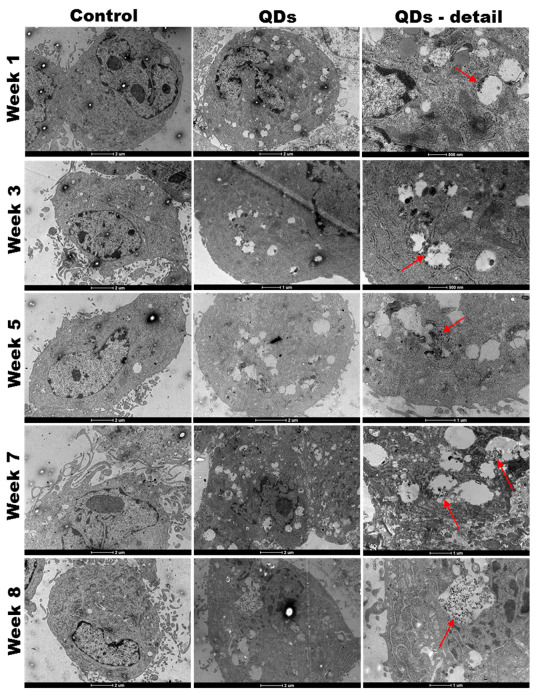
Ultrastructural appearance of MRC-5 human lung fibroblasts during the long-term exposure to Si/SiO_2_ QDs in comparison with control cells. Note the progressive accumulation of QDs within membrane-bound structures inside the cells as highlighted by the red arrows.

We observed by TEM analysis that QDs were taken up by the MRC-5 cells during the 8-week exposure as it can be observed in Figure 4. The internalized QDs appeared mainly as electron-dense clusters located inside cellular vesicles (red arrows in Figure 4), whose accumulation increased over the exposure time. It could be stated that large aggregates of QDs were internalized inside cells by macropinocytosis after cytoskeletal arrangements and membrane ruffling as described previously [20].

### 3.4. Effects of Long-Term Exposure to QDs of Autophagy Pathway in MRC-5 Cells

**Figure 5:**
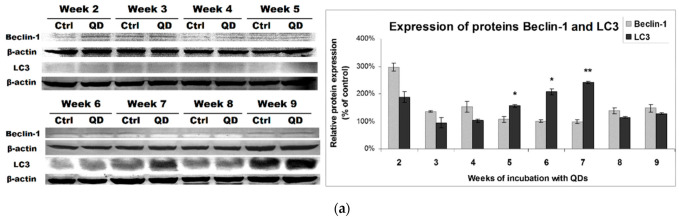

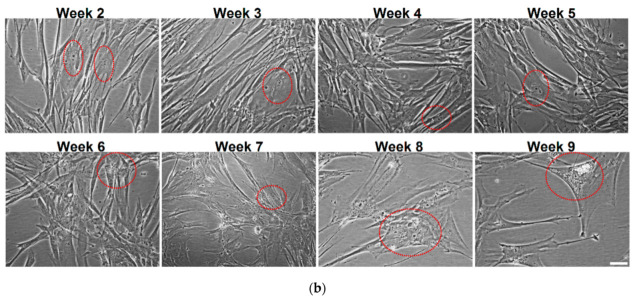
Effect of long-term exposure to Si/SiO_2_ QDs on the activation of autophagy pathway in MRC-5 cells. (**a**) Expression levels of Beclin-1 and LC3 proteins during the 9 weeks of incubation with Si/SiO_2_ QDs were calculated as mean ± SD (*n* = 3) and represented relative to control (* *p* < 0.05 and ** *p* < 0.01 compared to control). (**b**) Morphology of MRC-5 lung fibroblasts and progressive formation of autophagic vacuoles (red dot circles) were evidenced by phase-contrast optical microscopy during the 9 weeks of incubation with Si/SiO_2_ QDs. Scale bar of 50 µm is the same for all images.

Regarding autophagy, it is known that by the accumulation of autophagosomes and autophagic substrates, NPs can induce cell death due to unsustainable accumulation of autophagic vacuoles [28]. To investigate the ability of QDs to induce autophagy in MRC-5 cells during long-term exposure, the protein expression of Beclin-1 and LC3 was quantified (Figure 5a). Beclin-1 is a component of the phagophore nuclear complex involved in the early stages of autophagosome synthesis [29], while the LC3 protein is conjugated to phosphatidylethanolamine consequently to autophagy induction and inserted into the formation of the autophagosome’s membrane [30]. The most important increase in the Beclin-1 level, almost 3-fold that of the control, was noticed after the second week of exposure to QDs. After that, the protein level was much closer to that of the control, with only an increase of 50% above the control after the fourth and ninth weeks. Regarding the dynamics of LC3 protein expression during the 9-week incubation with QDs, there was an initial increase after the second week, followed by a recovery to the control levels during the next two weeks. By increasing the concentration of QDs in the culture medium, the cells responded through a progressive elevation of LC3 expression during the fifth, sixth and seventh weeks, then a return to the control was observed after the last two weeks.

Analysis of MRC-5 fibroblasts’ morphology using the phase-contrast light microscopy (Figure 5b) showed a progressive accumulation of perinuclear distributed vacuoles. This observation is also revealed by TEM in Figure 4. Further, in the last two weeks of incubation, numerous vacuoles were visualized, which could determine the appearance of cellular lesions.

The constitutive level of the Beclin-1 protein observed for weeks 5–7 under conditions of increased LC3 protein could be explained by the fact that LC3 can be overexpressed when intracellular protein aggregates are formed or, in this case, QDs aggregates sequestered in vacuoles (Figure 5b), independently of the induction of autophagy [30]. Most probably, the autophagosome formation started with the upregulation of Beclin-1 and LC-3 expressions at the end of week 2. The changes in expression levels observed during the next weeks could be explained by the initiation of an adaptive response by cells to QDs exposure. These results could point out the activation and modulation of a basal autophagy after week 3 as parts of the mechanism involved in the protection of cells from QDs cytotoxicity and also in sustaining the main functions in MRC-5 fibroblasts. This suggestion relies on the role of autophagy within the pro-survival processes following exposure to particles [31,32].

### 3.5. Effects of Long-Term Exposure to QDs of Apoptosis Pathways in MRC-5 cells

Among the main mechanisms of defense against oxidative stress is the activation of the Nrf2 signaling pathway, which controls the expression of genes whose protein products are involved in the detoxification and elimination of reactive species by conjugation reactions [33]. Upon activation, this protein is translocated into the nucleus. The regulation of the Nrf2 pathway is achieved through a complex mechanism, involving proteins such as NF-κB, p53, the AhR receptor for aromatic hydrocarbons and Notch1 [34].

**Figure 6:**
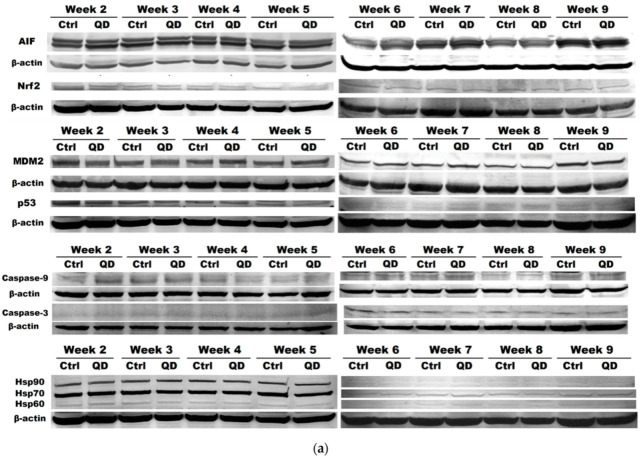

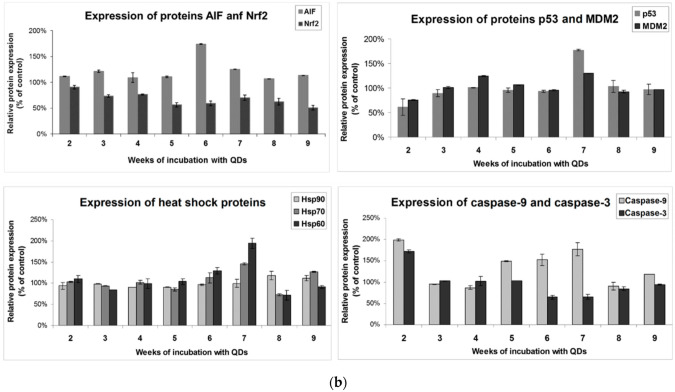
Effect of long-term exposure to Si/SiO_2_ QDs on the activation of apoptosis pathway in MRC-5 cells. (**a**) Protein expression levels of AIF, Nrf2, p53, MDM2, active caspases 9 and 3 and heat shock proteins (Hsp90, Hsp70 and Hsp60) during the 9 weeks of incubation with Si/SiO_2_ QDs were calculated as mean ± SD (*n* = 3) and (**b**) represented relative to control.

Therefore, protein expression of Nrf2 was followed as a cellular response to stress induced by exposure to Si/SiO_2_ QDs. As it can be seen in Figure 6, the level of Nrf2 protein was below the control one during the incubation with QDs, an observation that suggests that these NPs could induce destabilization and degradation of Nrf2 or could partially inhibit the synthesis of this protein. At the same time, the decreased expression of this protein, found in other nanotoxicity studies [35], could highlight a disturbance of redox homeostasis regulation in lung fibroblasts in the presence of QDs, given that the recovery of GSH by Nrf2 is critical for cell survival during oxidative stress [36].

The molecular mechanisms involved in controlling the balance between cell growth and death mainly include signaling pathways of apoptosis and autophagy. Therefore, besides the expressions of the factors involved in the autophagy pathway (Beclin-1 and LC3), the expressions of the proteins responsible for apoptosis activation (p53, MDM2, AIF, caspase-9 and caspase-3) and of heat shock proteins were investigated within this study.

The relatively low protein expression of p53 and its main regulator, MDM2, close to the control level, recorded during the first 6 weeks (Figure 6), suggested that the incubation of MRC-5 cells with a concentration of 5 μg/mL QDs did not lead to a p53 protein-mediated apoptotic response. However, the close levels of these two proteins could be due to the activation of p53 itself, given that ubiquitination by MDM2 causes the degradation of the protein constitutively present in the cell and p53 and MDM2 regulate each other. In this way, p53 stimulates the expression of MDM2, which in turn inhibits p53 activity through a negative feedback system developed at the level of protein degradation in the nuclear and cytoplasmic 26S proteasome and, through a positive feedback, promotes the mRNA translation of p53 through the interaction with it, striking a balance between p53 synthesis and degradation [37].

The increase in p53 protein expression observed after 7 weeks of incubation can be correlated with elevated levels of Hsp60 and Hsp70 proteins (Figure 6), which were activated to limit stress-induced lesions, facilitating cell recovery [38]. In particular, Hsp70 has an anti-apoptotic role in human cells, manifested primarily by binding to the AIF protein [39] which restricts its transport to the nucleus, by preventing Bax translocation from the cytoplasm to the mitochondria [40] and by preventing the formation of the apoptosome [41]. In parallel, Hsp60 exerts anti-apoptotic functions by binding to Bax and Bak proteins, preventing the initiation of the apoptotic mechanism and promoting cell survival [42]. At the same time, the lack of significant changes in Hsp protein expressions in the first 6 weeks of QDs exposure compared to the control highlighted the absence of cytotoxicity for these QDs concentrations that did not induce stress strong enough to stimulate Hsp expression in MRC-5 lung fibroblasts.

Caspase-3 plays an essential role in the terminal phase of apoptosis induced by various stimuli, and its expression varied during the QDs exposure. After starting at a level 75% higher than the control after the second week, the protein expression reached values close to the control ones which were maintained for the next 3 weeks, then it continued to decrease due to the increase in the concentration of QDs in the media. Although it has previously been shown that different NPs can induce cytotoxicity mediated by increasing the expression of caspases-3 and -9 [43], in this case, the level of caspase-3, an effector of apoptosis, decreased with increasing concentration of QDs in the last weeks of exposure, and expression of caspase-9 (which is an activator of caspase-3) remained elevated. A possible explanation for this observation could be the neutralization of caspase-3 activity by the inhibitor of apoptosis proteins (IAP), which recognize its active site and bind it by a short peptide sequence in a reverse orientation, preventing the entry of the substrate and, implicitly, its catalytic activity [44]. The increase observed in the eighth week in the expression of Hsp90, an inhibitor of Apaf-1 oligomerization [45] and of the formation of the active apoptosome, was reflected in the decrease in the amount of active caspase-9 because this is allosteric activated in the vicinity of the apoptosome [46].

Elevated expression of both Beclin-1 and LC3 proteins in the first 3 weeks correlates very well with the same profile obtained for caspases-3 and 9 (Figure 6), outlining the overall response of MRC-5 fibroblasts initiated in a first stage after the exposure to QDs began. Subsequently, the cells recovered from this shock of exposure to foreign particles that they did not recognize, with the expression levels of all determined proteins reaching the control.

In this context, it can be assumed that the increased level of caspase-3 generated after the initial concentration of QDs stimulated an apoptotic response, suggested by the similar result obtained for the active form of caspase-9. The increased expression of caspase-9 after weeks 5–7 could be correlated with the profiles obtained for the p53 protein and its regulator, MDM2, but also with the expression of Hsp and the AIF protein. At the same time, it can be noted that during the same incubation period, high values of LC3 were reached, which could suggest a critical point in the long-term exposure of MRC-5 cells to the Si/SiO_2_ QDs. These results regarding the decrease expression of proteins after weeks 8 and 9 compared to week 7 could appear as a consequence of the cell response changes due to an increase in the concentration of administered QDs (40 µg/mL). This aspect could appear delayed in terms of cell viability (Figure 3a) that showed a total decrease by 20% of the control at the end of week 9 and a reduction by 8% compared to week 7.

The AIF protein showed a slightly increased expression in the first 3 weeks of QDs exposure and then approached the level of the control (Figure 6). However, a significant increase was observed at the end of the sixth week, with the AIF level being brought back around the control after the last 2 weeks. This profile of AIF expression variation correlates with that of the p53 protein, but not with caspase-3 levels, given that AIF gene synthesis is regulated by basal p53 levels and, after its translocation to the nucleus, may participate in the process of apoptosis in a caspase-independent manner [47]. Further, it has been suggested that in the absence of apoptotic signals, constitutive induction of AIF (a mitochondrial protein) expression by p53 would indicate its cytoprotective role, maintaining optimal mitochondrial function [47], a mechanism that could be activated in case of exposure to QDs, taking into account the results obtained.

Furthermore, telomere defects are recognized by the cell as DNA damage, which induces the activation of the p53 protein, which in turn inhibits cell proliferation, inducing apoptosis and cell senescence [48]. Clearly, this theory has not been confirmed in the case of exposure to QDs, as the increase in p53 protein expression at the end of seventh week was not caused by lesions in the length of telomeric fragments as the values remained close to the control, but most probably due to the accumulation of ROS intrinsically generated by Si/SiO_2_ QDs.

## 4. Conclusions

The results of long-term exposure showed that doses of up to 40 μg/mL Si/SiO_2_ QDs triggered the activation of apoptotic and autophagy pathways, as well as the expression of molecules involved in the regulation of survival signaling pathways as an adaptive response to cellular stress in MRC-5 human lung fibroblasts. The increase in QDs concentration during the 9 weeks led to their internalization and accumulation of autophagic vacuoles, but no significant decrease in cell viability was observed in their presence. Further, in the range of tested concentrations, Si/SiO_2_ QDs did not exert genotoxicity by damaging the telomere sequences, as no shortening of the terminal restriction fragments was observed. These data provide experimental evidence to explain the low toxicity of Si/SiO_2_ QDs, but also sustain further investigation for these NPs as novel and promising tools for nanomedicine due to their high biocompatibility. Additionally, the results obtained with the concentrations used here and the repeated applications of two times per week could be relevant for a repeated human exposure to QDs of the personnel involved in manufacturing factories and medical staff.

## Data Availability

The data is available on the request from corresponding author.
